# Supplementary material for: Molecular Cluster Mining of Adrenocortical Carcinoma via Multi-Omics Data Analysis Aids Precise Clinical Therapy
Source: Cells. 2022 Nov 26;11(23):3784. doi: 10.3390/cells11233784 (PMC9737968; doi:10.3390/cells11233784)
Supplement: Supplementary file 1 [file cells-11-03784-s001.zip › cells-2019303-supplementary.pdf]

Supplementary Table S1. Summarization of clinical features of ACC subtypes in TCGA-ACC cohort.

**Parameters**	**level**	**ACC 1**	**ACC 2**	**ACC 3**	**P value**
number		32	22	24	
Age, years		48.53 ± 15.01	45.82 ± 19.17	45.12 ± 14.02	0.668
Status (%)	Alive	30 (93.8)	5 (22.7)	16 (66.7)	<0.001*
	Dead	2 (6.2)	17 (77.3)	8 (33.3)	
Overall survival (days, median [IQR])		1407.00 [898.00, 2464.50]	570.50 [490.00, 1174.00]	1541.00 [912.75, 2079.75]	<0.001*
Gender (%)	Female	18 (56.2)	13 (59.1)	16 (66.7)	0.727
	Male	14 (43.8)	9 (40.9)	8 (33.3)	
Stage (%)	Stage I	7 (21.9)	1 (4.5)	1 (4.2)	0.022*
	Stage II	20 (62.5)	7 (31.8)	10 (41.7)	
	Stage III	4 (12.5)	6 (27.3)	6 (25.0)	
	Stage IV	1 (3.1)	7 (31.8)	6 (25.0)	
	unknow	0 (0.0)	1 (4.5)	1 (4.2)	
Laterality (%)	Left	17 (53.1)	16 (72.7)	11 (45.8)	0.164
	Right	15 (46.9)	6 (27.3)	13 (54.2)	

*, P < 0.05

Supplementary Table S2. Summarization of clinical features of ACC subtypes in GEO cohort.

Parameters	level	ACC1	ACC2	ACC3	P value
number		32	34	23	
Age, years		45.91 ± 13.80	46.58 ± 19.35	46.79 ± 16.49	0.974
Status (%)	Alive	20 (62.5)	6 (17.6)	10 (43.5)	0.001*
	Dead	12 (37.5)	28 (82.4)	13 (56.5)	
Overall survival (days, median [IQR])		2914.00 [1799.75, 4427.75]	376.50 [229.00, 754.50]	1226.00 [632.00, 2330.00]	<0.001*
Gender (%)	Female	26 (81.2)	21 (61.8)	16 (69.6)	0.218
	Male	6 (18.8)	13 (38.2)	7 (30.4)	
Stage (%)	Stage I	3 (9.4)	0 (0.0)	4 (17.4)	0.031*
	Stage II	18 (56.2)	12 (35.3)	11 (47.8)	
	Stage III	2 (6.2)	3 (8.8)	1 (4.3)	
	Stage IV	4 (12.5)	16 (47.1)	5 (21.7)	
	unknow	5 (15.6)	3 (8.8)	2 (8.7)	
Side (%)	left	12 (37.5)	15 (44.1)	6 (26.1)	0.683
	right	10 (31.2)	11 (32.4)	10 (43.5)	
	unknow	10 (31.2)	8 (23.5)	7 (30.4)	

*, P < 0.05
